# Pharmacokinetics and Immunological Effects of Romidepsin in Rhesus Macaques

**DOI:** 10.3389/fimmu.2020.579158

**Published:** 2020-12-11

**Authors:** Adam J. Kleinman, Cuiling Xu, Mackenzie L. Cottrell, Ranjit Sivanandham, Egidio Brocca-Cofano, Tammy Dunsmore, Angela Kashuba, Ivona Pandrea, Cristian Apetrei

**Affiliations:** ^1^ Division of Infectious Diseases, Department of Medicine, School of Medicine, University of Pittsburgh, Pittsburgh, PA, United States; ^2^ Department of Pathology, School of Medicine, University of Pittsburgh, Pittsburgh, PA, United States; ^3^ University of North Carolina Eshelman School of Pharmacy, University of North Carolina, Chapel Hill, NC, United States; ^4^ Department of Immunology, School of Medicine, University of Pittsburgh, Pittsburgh, PA, United States; ^5^ Department of Infectious Diseases and Immunology, School of Public Health, University of Pittsburgh, Pittsburgh, PA, United States

**Keywords:** romidepsin, histone deacetylase inhibitors, latency reversing agents, human immunodeficiency virus, simian immunodeficiency virus, HIV latency, pharmacokinetics

## Abstract

HIV/SIV persistence in latent reservoirs requires lifelong antiretroviral treatment and calls for effective cure strategies. Romidepsin (RMD), a histone deacetylase inhibitor, was reported to reactivate HIV/SIV from reservoirs in virus-suppressed individuals. We characterized in detail the pharmacokinetics and safety profile of RMD in three SIV-naïve rhesus macaques which received two rounds of treatment. In plasma, RMD mean terminal half-life was 15.3 h. In comparison, RMD mean terminal half-life was much longer in tissues: 110 h in the lymph nodes (LNs) and 28 h in gastrointestinal tract. RMD administration was accompanied by transient liver and systemic toxicity. Isoflurane anesthesia induced near-immediate transient lymphopenia, which was further exacerbated and extended with the extensive immune modifications by RMD. The effect of RMD on circulating immune cells was complex: (i) slight increase in lymphocyte death rates; (ii) transient, robust increase in neutrophils; (iii) massive downregulation of lymphocyte surface markers; (iv) important migration of CD3^+^ T cells to the gut and LNs; and (v) hindrance to CD8^+^ T cell functionality, yet without reaching significance. Our results show that, in contrast to transient plasma concentrations, RMD has a long-term presence in tissues, with multiple immunomodulatory effects and minimal to moderate kidney, liver, and lymphocyte toxicities. As such, we concluded that RMD can be used for “shock and kill” approaches, preferentially in combination with other latency reversal agents or cytotoxic T lymphocyte boosting strategies with consideration taken for adverse effects.

## Introduction

The advent of antiretroviral therapy (ART) has been a tremendous success in extending the life expectancy of persons living with human immunodeficiency virus (HIV) (1). However, ART is virostatic and does not target the integrated virus, which persists in latent reservoirs. As such, an HIV cure is needed (2). The proof of concept that HIV cure is possible was provided by the “Berlin patient,” who received an allogeneic stem cell transplantation using donors homozygous for the *CCR5 Δ32* allele (3) and was in remission off ART for over 10 years (4). A second patient that underwent a similar procedure (the “London patient”) is also reported to be in remission (5). However, attempts to reproduce this clinical outcome *via* stem cell transplantation from donors with functional CCR5 genes, or very early ART initiation have been unsuccessful and resulted in viral rebound 3–48 months after ART cessation (6–8). These failures are due to the persistence of the latent HIV reservoirs which, upon ART cessation, can reactivate and drive a productive infection (9–12). Studies of the early dynamics of the simian immunodeficiency virus (SIV) reservoir in nonhuman primates (NHPs) showed that ART initiation as early as 3 days postinfection, i.e., prior to detectable viremia, did not prevent reservoir seeding (13).

Not only is the reservoir very rapidly established, but it is also proteiform. Numerous cellular types are able to host latent HIV/SIV and contribute to the reservoirs: central memory (14–16), transitional memory (15, 16), follicular T helper CD4^+^ cells (17), stem cell memory T cells (18), and regulatory T cells (19, 20). The common feature of these cells is that they are of resting phenotype (2, 9, 10, 21–24), and that no marker can clearly identify the latently infected cells (25), which makes interventions toward reservoir eradication and HIV cure extremely difficult (20).

One of the most popular HIV cure strategies is the “shock and kill,” the goal of which is to induce viral transcription from the latent reservoir using latency reversing agents (LRAs), followed by immune-mediated clearance of infected cells, thus depleting the viral reservoir, in the presence of ART to prevent *de novo* infections of uninfected cells (26–29). Histone modifications around the integrated proviral HIV long-terminal repeats (LTRs), stemming from histone deacetylase activity, inhibit transcription, and lead to viral latency (30–33). Thus, histone deacetylase inhibitors (HDACi) loosen the DNA around histones and free the provirus for transcription and viral production (34). This mechanism makes HDACi one of the most studied classes of drugs for the “shock and kill” approach (35–37).

Of the HDACi tested for HIV latency reversal, the depsipeptide romidepsin (RMD) (38, 39) has been extensively studied. It has been shown to be the most potent HDACi in terms of HIV reactivation, both *in vitro* and *ex vivo* (40). RMD reactivates latent SIV with subsequent boosts in T cell activation (41, 42) in rhesus macaques (RMs) and induces reactivation of latent HIV in humans (43, 44). There is debate as to whether RMD has an impact on the cytotoxic T lymphocyte (CTL) response to viral antigens, with some studies finding little change (41, 43), and another demonstrating inhibition of the CTL response (45). This is of particular importance, as the cell-mediated immune response is a major player in controlling virus. In view of this relatively moderate success, we performed a detailed assessment of RMD pharmacokinetics in plasma and tissues, its toxicity and tolerability, and its impact on the counts and function of immune cell populations from circulation, lymph nodes (LNs), and intestines in SIV-naïve RMs to determine whether the immunological effects of RMD are appropriate for use as an HIV therapeutic.

## Materials and Methods

### Ethics Statement

All the RMs included in this study were housed and handled at the University of Pittsburgh following the standards of the Association for Assessment and Accreditation of Laboratory Animal Care (AAALAC) and the Animal Welfare Act (46). The University of Pittsburgh approved these experiments under the Institutional Animal Care and Use Committee (IACUC) protocol 15045866.

### Study Design

**Figure 1:**
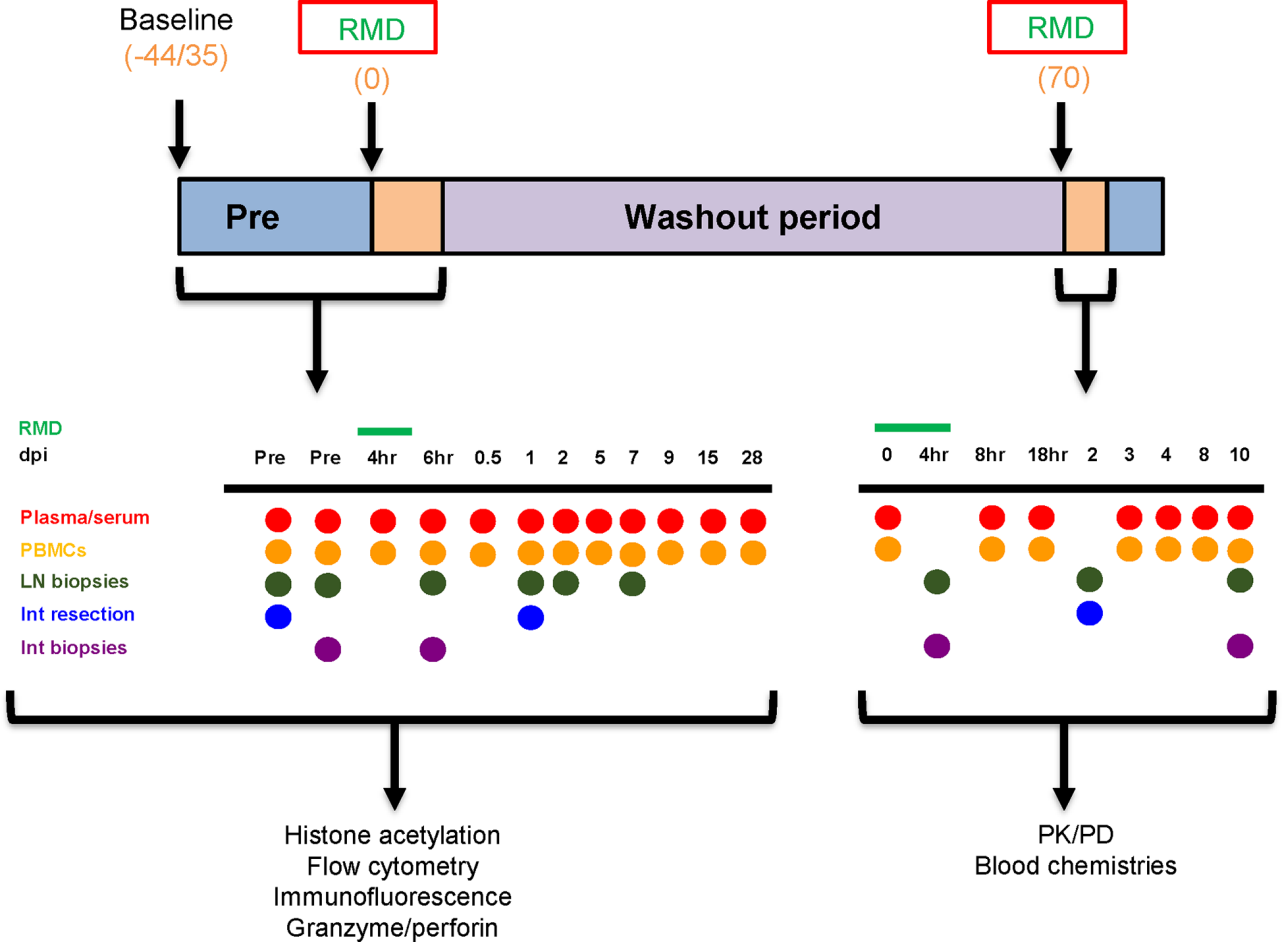
Experimental design. The timeline denotes the days at which baseline measurements began, romidepsin (RMD) was administered, and the washout period, in days postinfusion. The sampling schedules for both the first and second rounds of RMD treatment are shown. Blood chemistries, flow cytometry, immunohistochemistry, histone acetylation, and granzyme/perforin assays were completed with the samples taken from the first round of RMD treatment. PK/PD of RMD in blood and tissues was assessed for the second round of RMD treatment. dpi, days postinfusion; PBMCs, peripheral blood mononuclear cells; LN, lymph node; Int, intestinal.

Six RMs (*Macaca mulatta*) were included in this study. Three SIV-naïve RMs were treated twice with 7 mg/m^2^ of RMD administered over a 4-h IV infusion and separated by a 70-day washout period to allow for complete clearance of RMD. Two rounds were utilized to gather additional cells which were limited by the extensive sampling conducted at each treatment. Three additional RMs received infusions with saline solution in the same conditions of sedation and restraint as RMs in the RMD group (control group). Sampling was performed as shown (**Figure 1**). Both superficial and mesenteric LNs were biopsied along with intestinal resections. Tissues were not collected from the control RMs.

### Animals, Treatments, and Sampling

Two male and one female SIV-naïve RMs ages 2 to 5 years and weighing between 5.6 and 8.9 kg, received RMD (Istodax, Celgene Corporation, Summit, NJ) at a dose of 7 mg/m^2^. RMD was administered by slow infusion *via* catheter for 4 h. The animals were sedated with ketamine (10–20 mg/kg). An IV catheter was placed in a saphenous or cephalic vein. An endotracheal tube was inserted and maintained until the animals were ready to be placed back into their cage, with 1%–2% isoflurane to keep them anesthetized. Blood pressure, oxygen, heart, and respiratory rates were measured every 15 min. Animals were maintained in the surgical plane until after the 6- or 8-h postinfusion bleeds to reduce sedation events.

To assess the impact of this prolonged anesthesia procedure on the tested immune and toxicity parameter, two additional male and one female RMs, aged 17–18 years and weighing between 11.7 and 13.3 kg, were included as controls and were subjected to the same anesthesia and infusion procedures as the RMD treated RMs, but RMD was replaced with a 0.85% saline solution. These animals were bled immediately prior to infusion and then at timepoints of 6 h, and 1, 2, 5, and 7 days postinfusion.

RMD was given in two separate rounds with a 70-day interval to allow for complete washout. The dose was established based on previous studies in humans (47, 48) and our previous experience with RMD (41), which showed efficacy of RMD in RMs with relatively limited biological side effects.

**Figure 1** illustrates the sampling schedule. Briefly, during the first RMD treatment, sampling included two bleeds at 35 and 13 days prior to treatment, four blood draws within 1 days postinfusion (4, 8, 16, and 24 h postinfusion), and then at 2, 5, 7, 9, 15, and 28 days postinfusion. Intestinal biopsies were taken at 13 days prior to infusion; intestinal resections were taken at 30 days before infusion and 1 day postinfusion; LNs were collected at 30 and 13 days prior to infusion, 6 h postinfusion, and 1 and 7 days postinfusion. The samples collected during the first round of RMD treatment were used for immune population dynamics and functionality.

During the second round of RMD treatment, blood was taken at 0, 2, 4, 6, and 18 h postinfusion, and at 3, 4, 8, and 10 days postinfusion. Intestinal resections from the jejunum were taken at 2 days postinfusion. Intestinal biopsies were taken at 4 h postinfusion and 10 days postinfusion. Superficial LNs were taken at 4 h postinfusion and 10 days postinfusion. Mesenteric LNs were collected at 2 days postinfusion. The samples collected during the second round were utilized for the pharmacokinetic assays and toxicity assessment.

Blood was collected intravenously from the femoral vein. Animals were sedated with 10–20 mg/kg ketamine IM with assistance from a squeeze cage. The site of collection was wiped with alcohol and after retraction of the collection needle, the site of collection was compressed until the vein clotted.

Complete blood counts (CBCs) and serum chemistries were monitored in the collected samples from both the RMD-treated RMs and control RMs by either the Marshfield Laboratories (Cleveland, OH), IDEXX Reference Laboratories (IDEXX Laboratories, Inc, Westbrook, ME) or in house, using a ProCyte Hematology Analyzer (IDEXX Laboratories, Inc).

Endoscope-guided intestinal biopsies were collected at the timepoints outlined in **Figure 1**. Animals were sedated with 10 mg/kg ketamine for this procedure. To collect adequate numbers of intestinal lymphocytes for analyses, between 12 and 15 pinch biopsies (approximately 1 mm^3^ each) of intestinal mucosa (duodenum and upper jejunum) were obtained at each timepoint using an endoscope and small biopsy forceps.

Superficial LNs were collected from each RM at the times indicated in **Figure 1**. The animals were prepared aseptically using standard procedures (i.e., hair shaved from surgical site and site cleaned with alcohol and betadine) and draped using sterile drapes. The superficial LNs were located by palpation and the overlying skin incised. The LN was freed and removed by blunt dissection and ligation of attached vessels. Soft tissue was sutured with 3-0 Vicryl. Skin was sutured with a subcuticular pattern using 3-0 Vicryl and/or skin glue.

Jejunal resection, anastomoses, and mesenteric LN biopsies were conducted at the times indicated in **Figure 1**. Each animal was prepared for surgery using standard procedures and draped using sterile drapes. An intravenous catheter was placed in the saphenous or cephalic veins and animals were intubated and maintained at a surgical plane of anesthesia using medical grade oxygen and isoflurane. A ventral midline incision was made and the jejunum isolated and packed off from the rest of the abdominal contents. After clamping the jejunum, a 20-cm section of jejunum was resected, and the anastomoses performed using 4-0 PDS in a simple-interrupted pattern. The rent in the mesentery was closed with 3-0 Vicryl in a simple continuous pattern. Mesenteric LNs were removed following isolation and ligation of any connected blood vessels. The incision was closed using 3-0 Vicryl in a simple continuous pattern in the muscle layer and the skin closed with a subcuticular pattern using 3-0 Vicryl followed by application of tissue glue along the incision line.

### Cell Separation From Whole Blood

Peripheral blood mononuclear cells (PBMCs) were separated from whole blood as described (49, 50), and either used in an assay immediately or frozen. Briefly, whole blood was centrifuged at 2,200 rpm for 20 min and plasma was collected. The blood was then layered over lymphocyte separation media (LSM, MPBIO, Solon, OH) and separated by centrifugation at 2,200 rpm for 20 min. The buffy coat containing PBMCs was collected, washed with 1× phosphate-buffered saline (1× PBS, Lonza, Basel, Switzerland), and counted. PBMCs were frozen at 5 million cells/ml with freezing media consisting of 95% fetal bovine serum (FBS, VWR, Radnor, PA, USA) and 5% DMSO (Thermo Fisher Scientific, Walthead, MA).

### RMD Pharmacokinetics

RMD was quantified in both plasma and tissues using LC-MS/MS. Tissue biopsies were weighed then homogenized in Precellys^®^ hard tissue grinding kit tubes (Cayman Chemicak, MI, USA) with 1 ml of methanol. Plasma and tissue homogenates were then extracted by protein precipitation with isotopically labeled internal standards (atazanavir-d5 and darunavir-d9 for tissue and plasma, respectively). Analytes were separated by reverse phase chromatography on an Atlantis T3 (50 × 2.1 mm, 3 μm) analytical column (Waters, Milford, MA, USA) prior to detection on an API-5000 triple quadrupole mass spectrometer (AB SCIEX, Foster City, CA, USA). Calibration standards and quality control samples were within 20% of nominal values with a dynamic range of 0.02–50 ng/ml of tissue homogenate and 0.2–200 ng/ml of plasma. Tissue homogenate concentrations were normalized for sample weight assuming a tissue density of 1 g/ml. Noncompartmental analysis was performed using Phoenix WinNonlin v8.1 software (Pharsight Cooperation, Cary, NC, USA) to calculate the area under the concentration time curve (AUC_0-10days_) using the linear trapezoidal rule. To calculate the half-life of RMD in plasma, the terminal elimination rate constant (kel) was estimated by fitting a linear regression line on a semi-log plot to the individual plasma concentration vs. time data. All plasma observations after 96 h were below the limit of quantification and omitted from the fitting. Given the relatively sparse sampling strategy in tissues, the mean concentration time profile was used to estimate kel as described above.

### Flow Cytometry

Whole blood was stained to monitor the impact of RMD on the immune cell populations. TruCount was used to determine absolute counts of CD3^+^, CD4^+^, CD8^+^ T cells and CD20^+^ B cells as described (51–53). Fifty microliters of whole blood were stained with antibodies against CD3-V450, CD4-APC, CD20-APC-H7, and CD45-PerCP in TruCount tubes (BD Biosciences, Franklin Lakes, NJ, USA) with a set number of fluorescent beads as internal standards. CD8^+^ T cell counts were assessed through the ratio of CD8^+^ to CD3^+^ T cells. Whole blood was also stained with combinations of the following fluorescently labeled antibodies: CD3-V450 (SP34-2), CD4-APC (L200), CD8-PE-CF594 (RPA-T8), CD14-PE-Cy7 (M5E2), CD38-FITC (AT-1) (Stemcell, Vancouver, BC, CA), CD20-APC-H7 (2H7), CD69-APC-Cy7 (FN50), CD95-FITC (DX2), CCR5-PE (3A9), Annexin V-FITC, HLA-DR-PE-Cy7 (L243), NKG2A-PE (Z199) (Beckman Coulter, Pasadena, CA), Strepavidin-Alexa Fluor 750 (Life Technologies, Carlsbad, CA), Biotin-CCR7 (eBioscience, San Diego, CA), LIVE/DEAD Fixable Blue Dead Cell Stain Kit (Thermo Fisher Scientific); all antibodies were from BD Biosciences unless otherwise noted. For Ki-67-PE (B56) intracellular staining, cells were fixed and permeabilized prior to Ki-67 staining. Data were acquired with a LSR-II flow cytometer (BD Biosciences) or Fortessa flow cytometer (BD Biosciences) and analyzed with FlowJo software 10.7.0 (Treestar, Ashland, OR), as described (51, 54).

### T Lymphocyte Functional Assay

To simplify the complication of RMD treatment and SIV infection, it was vital to assess the impact of RMD administration on the T-cell function, particularly on the cellular immune responses on uninfected RMs. Due to the incapability to assess the SIV-specific immune responses, the overall cytotoxic capacity of lymphocytes in the blood was assessed as follows. Frozen PBMCs were thawed, counted, and were either unstimulated or stimulated with PMA and ionomycin (Sigma, St. Louis, MO). PBMCs were stained with the following antibodies: CD3-V500 (SP34-2), CD4-APC (L200), CD8-PE-CF594 (RPA-T8), CD107a-APC-Cy7 (H4A3), Granzyme A-PerCP/Cy5.5 (CB9) (BioLegend), Granzyme B-Alexa Flour 700 (GB11), Granzyme K-FITC (GM6C3) (Santa Cruz Biotechnology, Dallas, TX), IFN-γ-FITC (4S.B3), and Perforin-PE (B-D48) (BioLegend); antibodies were from BD Biosciences unless otherwise noted. Stained PBMCs were then acquired on an LSR-II flow cytometer. Data were analyzed using FlowJo software (Treestar).

### Histone Acetylation Assay

The efficacy of RMD treatment was assessed by monitoring histone acetylation of frozen PBMCs: at the baseline, 6 h postinfusion, and 1, 2, and 5 days postinfusion, using a flow-cytometrical method, as described (41). Briefly, separated PBMCs were stained for surface markers for 20 min with: CD3-APC-Cy7 (SP34-2), CD4-V500 (L200), CD8-PE (SK1), CD14-BV570 (M5E2) (BioLegend, San Diego, CA), CD28-PE-CF594 (CD28.2), CD69-BV421 (FN50), CD95-PE-Cy5 (DX2), PD-1-PE-Cy7 (EH12.2H7) (BioLegend); all antibodies were from BD Biosciences, unless otherwise stated. PBMCs were then lysed with PhosFlow Lyse/Fix buffer (BD Biosciences) for 30 min and permeabilized with Perm Buffer [0.4% Triton X-100 in PBA (Sigma)] for 10 min, followed by washing. Permeabilized PBMCs were stained with the following antibodies for intracellular markers: Ac-H4 (3HH4-2C2) (Active Motif, Carlsbad, CA, USA) and Ki-67-Alexa Flour 647 (BD Biosciences). Ac-H4 recognizes motifs on histones H3 and H4 and was conjugated to FITC using Zenon reagent kit (Invitrogen, Carlsbad, CA). Stained PBMCs were then washed, fixed with BD Stabilizing Fixative (BD Biosciences), and acquired with a LSR-II flow cytometer. Data were analyzed with FlowJo software.

### *In Vitro* Assessment of RMD Impact on Immune Cells and Homing Markers

To assess the RMD impact on the expression of cellular and homing markers, fresh PBMCs collected from 3 SIV-naïve RMs were subjected to either no RMD treatment or escalating doses of RMD (2, 10, and 20 ng/ml). The PBMCs were incubated with RMD in RPMI1640 supplemented with 1% Penicillin/Streptomycin, 1% HEPES buffer, 1% L-glutamine, and 5% heat-inactivated bovine calf serum for 0, 1, 2, or 5 days. PBMCs were then stained with CD3-V500, CD4-Alexa Flour 700 (L200), CD8-PE-CF594, CCR4-PE-Cy7 (1G1), CCR5-APC (3AP), CCR7-PE (3D12), CCR9-FITC (112509) (R&D Systems, Minneapolis, MN, USA), β7-PE-Cy5 (FIB504), and LIVE/DEAD Fixable Blue Dead Cell Stain Kit (Thermo Fisher Scientific); all antibodies were from BD Biosciences unless otherwise noted. Stained PBMCs were acquired on a LSR-II and data were analyzed with FlowJo software (TreeStar).

### Immunofluorescence

CD3^+^ T cell frequency in the intestine and LNs was assessed through immunofluorescence (IF), as described (55). Fixed, paraffin-embedded tissues from the three RMs were cut and placed onto microscope slides. The slides were treated three times with xylene (Thermo Fisher Scientific), run through an ethanol battery, washed with dH_2_O, and placed into 1× PBS (Thermo Fisher Scientific). Slides were microwaved with Antigen Unmasking Solution (Vector Labs, Burlingame, CA) for antigen retrieval. Once slides had cooled, they were washed three times in 1× PBS and the tissues were circled with Immedge Pen (Vector Labs) to create a hydrophobic barrier. Dako Serum-Free Protein Block (Agilent, Santa Clara, CA) was added to the tissues. The Dako Protein Block was dumped off and the slides incubated with mouse α-CD3 (Agilent). The primary antibody was dumped off and slides washed in 1× PBS as previously noted. The remaining steps were completed in the dark. The slides were incubated with the secondary antibody, goat α-mouse (Alexa Fluor 488) (Invitrogen) followed by a subsequent 1× PBS wash. The slides were incubated with DAPI (Millipore Sigma, Burlington, MA), and then dumped off and washed with 1× PBS, followed by dH_2_O, and dried. Coverslips were then mounted to the slides with fluorescent mounting media (Agilent). Slides were visualized and imaged with Zeiss Imager M1 microscope and Axiovision software [V4.8.2.0 (Carl Zeiss AG, Oberkochen, Germany)]. Image quantifications were performed using the FIJI distribution (56) of ImageJ 1.52c (57), where positive fluorescent signals were isolated by setting color thresholds and the percent positive area was calculated.

### Statistics and Data Analysis

**Figure 2:**
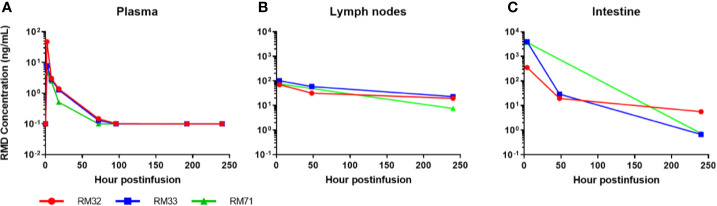
Romidepsin concentrations in plasma and tissue post 4-h infusion of 7 mg/m^2^. RMD concentrations of 3 SIV-naïve RMs in plasma **(A)**, lymph node **(B)**, and intestinal tissue **(C)**.

**Figure 3:**
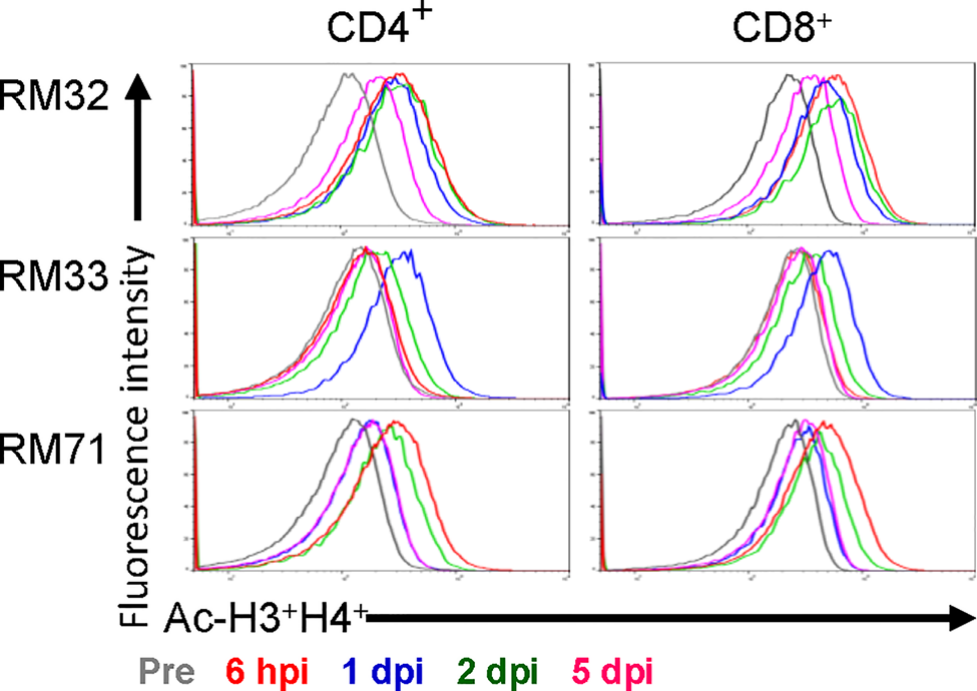
Histone acetylation of peripheral CD4^+^ and CD8^+^ T cells with RMD treatment. The levels of histone acetylation (H3 and H4) were measured in peripheral CD4^+^ and CD8^+^ T cells by flow cytometry prior to RMD treatment, at 6 hpi, 1, 2, and 5 dpi.

**Figure 4:**
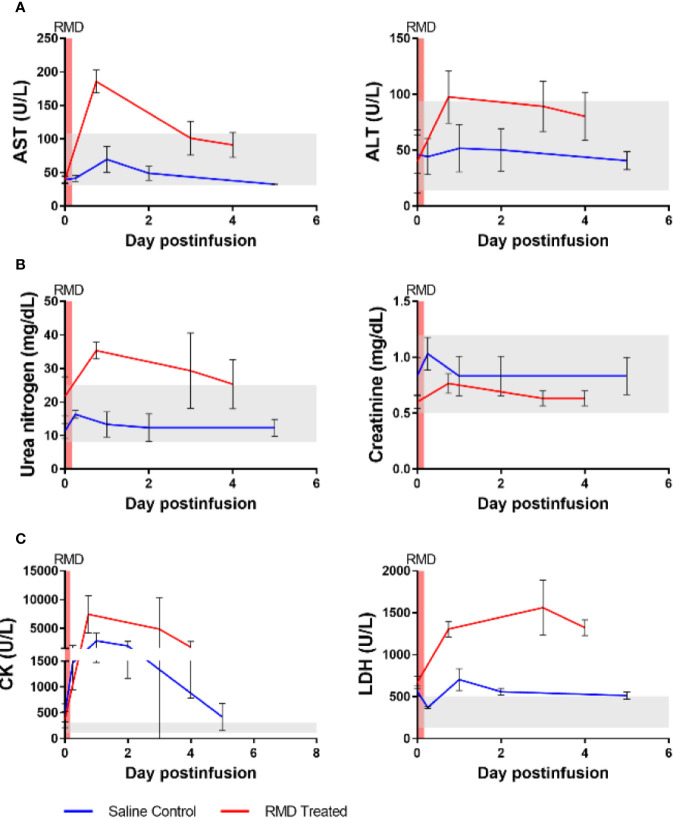
Blood chemistries of markers for hepato- and nephrotoxicity and general markers of cell death of three SIV-naïve rhesus macaques after second round of RMD. **(A)** Hepatotoxicity markers, aspartate aminotransferase (AST) and alanine transaminase (ALT). **(B)** Nephrotoxicity markers, urea nitrogen and creatinine. **(C)** General markers of cell death, creatinine kinase (CK) and lactate dehydrogenase (LDH). The grey area represents the average reference values for the markers in three SIV-naïve, untreated RMs.

**Figure 5:**
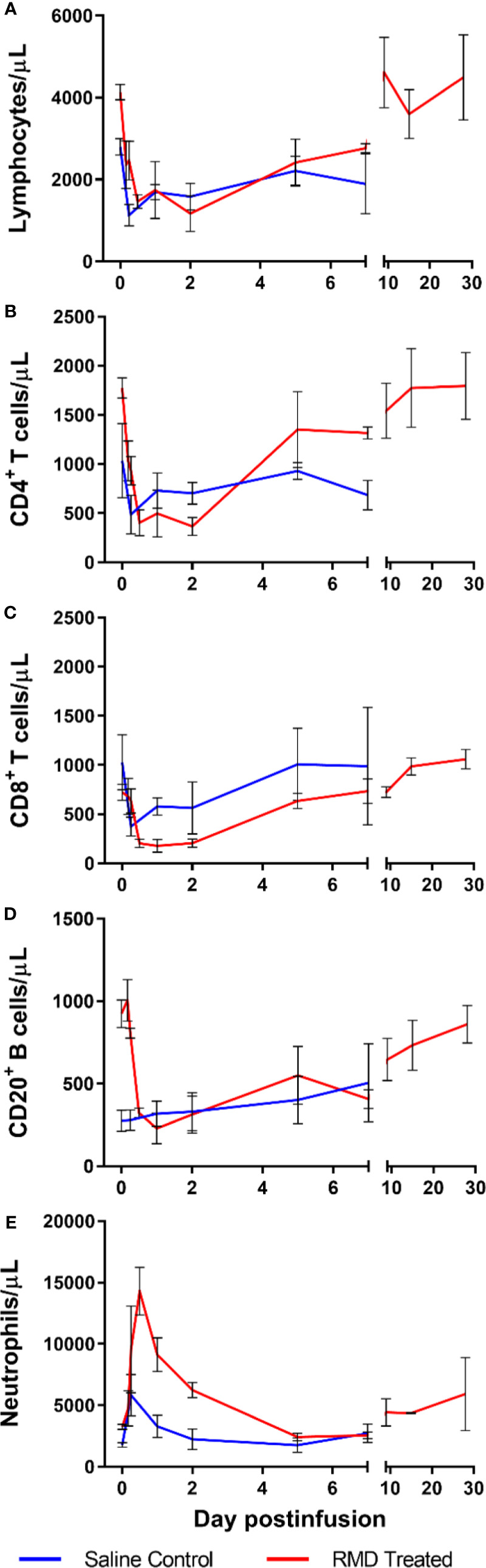
Immune cell populations in 3 SIV-naïve RMs treated with 7 mg/m^2^ RMD *via* 4-h infusion vs. 3 saline-treated controls. Total lymphocyte counts **(A)**, absolute counts of CD4^+^ **(B)** and CD8^+^ **(C)** T cells, CD3^−^CD20^+^ B cells **(D)**, and segmented neutrophils **(E)** from circulation.

**Figure 6:**
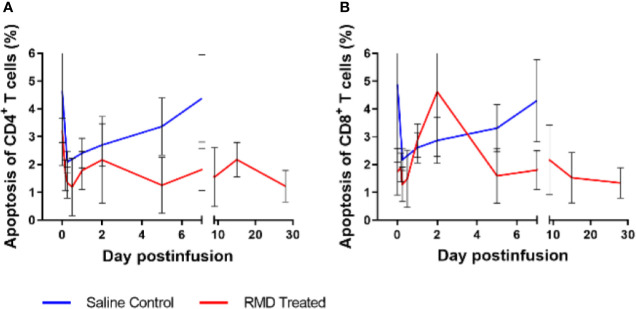
Peripheral blood lymphocyte apoptosis with RMD treatment. Whole blood was stained with Annexin V and Live/Dead stain to determine apoptosis of CD4^+^ **(A)** and CD8^+^ **(B)** T cells; animal number, n = 3 per group.

**Figure 7:**
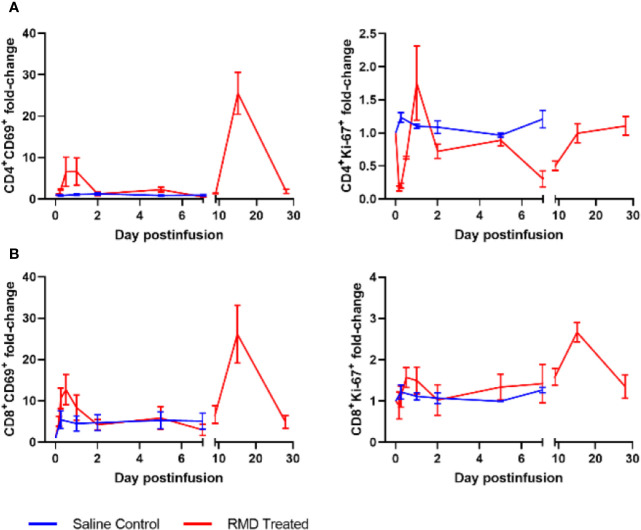
Lymphocyte activation with RMD treatment. Whole blood was stained for **(A)** CD4^+^ and **(B)** CD8^+^ lymphocyte markers: CD69 and Ki-67; animal number, n = 3 per group.

**Figure 8:**
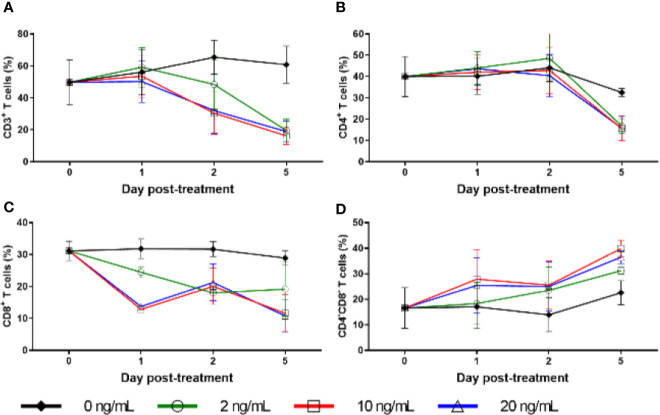
Cell surface marker expression with RMD treatment *in vitro*. Percentage of freshly separated PBMCs expressing CD3 **(A)**, CD4 **(B)**, and CD8 **(C)**, and CD4^−^CD8^−^ double negative PBMCs **(D)** treated with increasing RMD doses of (0, 2, 10, and 20 ng/ml); animal number: n = 3.

**Figure 9:**
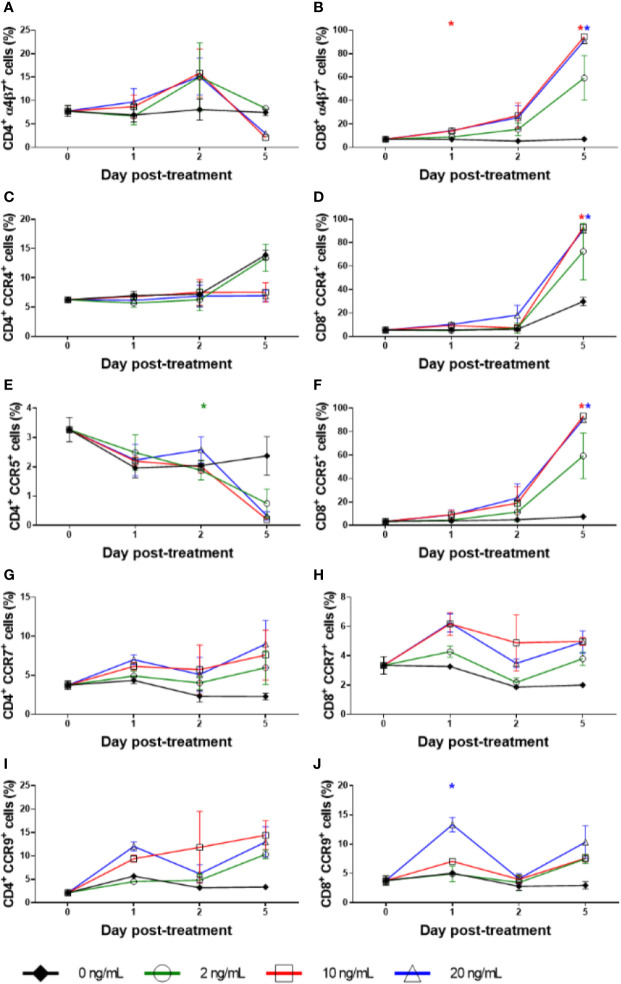
Homing marker expression with RMD treatment *in vitro*. The expression percentage of α4β7^+^ on **(A)** CD4^+^ and **(B)** CD8^+^, CCR4^+^ on **(C)** CD4^+^ and **(D)** CD8^+^, CCR5^+^ on **(E)** CD4^+^ and **(F)** CD8^+^, CCR7^+^ on **(G)** CD4^+^ and **(H)** CD8^+^, and CCR9^+^ on **(I)** CD4^+^ and **(J)** CD8^+^ with increasing dosages of RMD (0, 2, 10, and 20 ng/ml); animal number: n = 3. Asterisks indicate statistical significance of means to baseline as shown with concentration color (p < 0.05).

**Figure 10:**
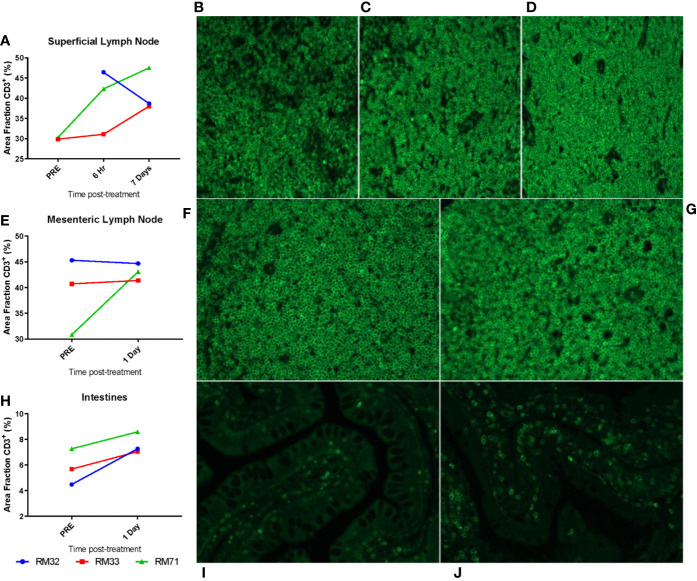
Migration of CD3^+^ T cells to tissues from the periphery. Superficial LNs **(A**–**D)**, mesenteric LNs **(E–G)**, and intestinal resections **(H**–**J)** were embedded in paraffin and subsequently stained for CD3. Threshold quantification of total positive area for CD3 fluorescence for superficial LNs **(A)**, mesenteric LNs **(E)**, and intestinal resections **(H)**. Representative images from superficial LNs are shown at pre **(B**, 6 hpi **(C)**, and 7 dpi **(D)**; mesenteric LNs at pre **(F)**, 1 dpi **(G)**; intestinal resections at pre **(I)** and 1 dpi **(J)**.

**Figure 11:**
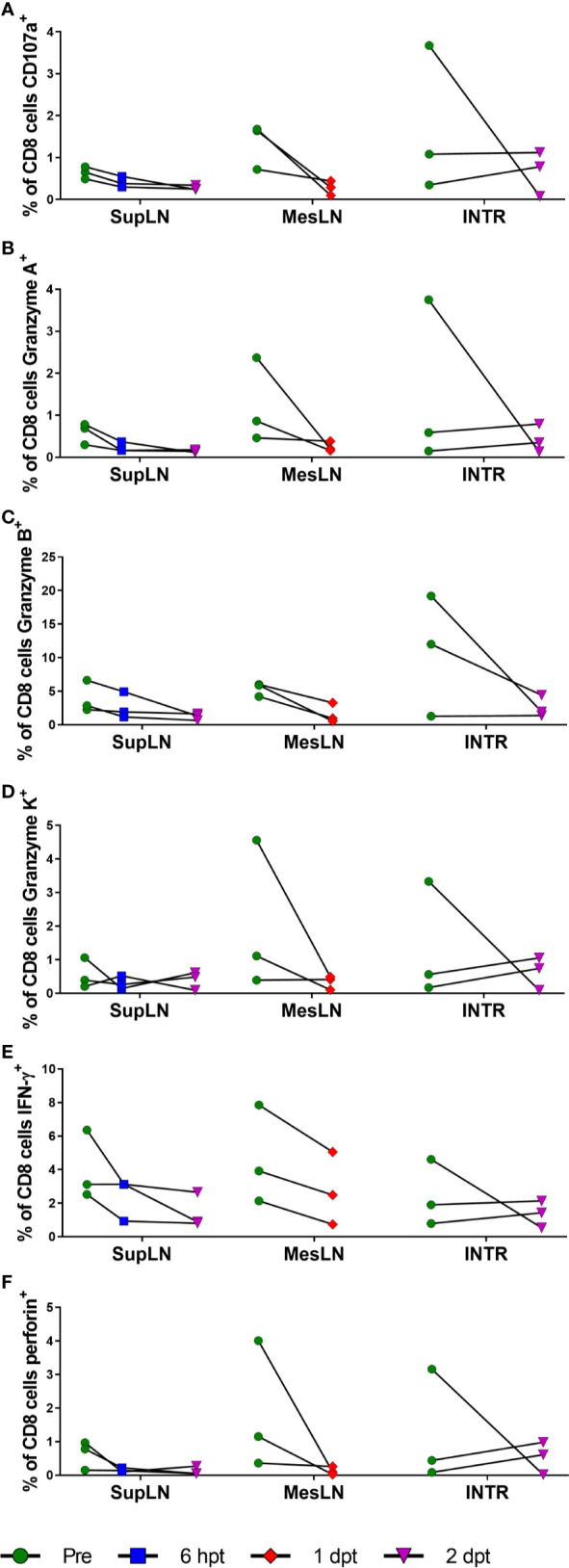
Cytotoxic T lymphocyte function with RMD treatment. Functional assays were performed for cytotoxic T lymphocyte markers of functionality, CD107a **(A)**, Granzyme A **(B)**, Granzyme B **(C)**, Granzyme K **(D)**, IFN-γ **(E)**, and perforin **(F)** in CD8^+^ T cells stimulated with PMA and ionomycin. SupLN, Superficial LN; MesLN, Mesenteric LN; INTR, Intestinal resections.

Graphing and statistical analyses were completed with Prism 8.4.3. Data were expressed as individual values in **Figures 1**–**7**, **10**, and **11**. Data for **Figures 8**, **9**, and **S1** were expressed as means ± standard errors of the means (SEM). To compare differences in toxicities and lymphocyte dynamics, a Friedman test with Dunn’s multiple comparisons test was utilized. To analyze cell surface marker expression and homing marker expression, Mixed-effects model with Geisser-Greenhouse correction and Tukey’s multiple comparisons test was used. CD3^+^ cell migration to tissue sites and CTL functionality with RMD treatment utilized Wilcoxon paired non-parametric (two-tails) test was used. For all statistical tests p < 0.05 was considered significant.

## Results

### RMD Distributes Extensively Into the Mucosal Tissues and Persists for at Least 10 Days

RMD was quantified in plasma, LNs, and the intestine after the second IV infusion. As expected, concentrations were the highest in the plasma at the first sampling time (2 h postinfusion: 4.7, 7.7, and 47 ng/ml). The mean terminal half-life of RMD in the plasma was 15.3 h with quantifiable concentrations for up to 3 to 4 days postinfusion (**Figure 2A**). Assuming a tissue density conversion of 1.0 g/ml according to previous publications (58–60), the concentrations in LNs were the highest (70, 75, and 101 ng/ml) at 4 h postinfusion and the mean terminal half-life (110 h) was seven-fold longer than plasma with quantifiable concentrations for 10 days postinfusion (**Figure 2B**). In the GI tract, RMD concentrations were the highest [median (range) of 3,665 (354–3,932) ng/g] at 4 h postinfusion, and the mean terminal half-life (28 h) was 1.8-fold longer than plasma with quantifiable concentrations for 10 days postinfusion (**Figure 2C**). RMD exposure (i.e., area under the concentration vs. time curve; AUC_0-10days_) was 27–153-fold higher in LNs and 426–2,860-fold higher in intestine than in plasma.

To assess the functional impact of RMD, we monitored the levels of H3 and H4 histone acetylation within CD4^+^ and CD8^+^ T cells from blood. Histone acetylation increased rapidly in RMs upon RMD administration (**Figure 3**), peaking at 6 h postinfusion in two of three RMs and 1 day postinfusion in the third RM (RM33). The histone acetylation returned to near baseline level by 5 days postinfusion in both CD4^+^ and CD8^+^ T cells in all of the RMs (**Figure 3**), similar to previous findings at the same dosage (41), and agreeing with the drug concentrations in plasma (**Figure 2A**).

### Transient Toxicity With RMD Treatment

We next assessed RMD toxicity by monitoring multiple serum biomarkers. Potential hepatotoxicity was documented by postinfusion increases of aspartate aminotransferase (AST) (186 U/L at 18 h postinfusion) and alanine aminotransferase (ALT) (98 U/L at 18 h postinfusion) (**Figure 4A**), both of which surpassed the normal ranges of AST (31-108 U/L) and ALT (14-94 U/L). The increase in AST was significant relative to pretreatment baseline levels (39 U/L) (p < 0.05) while the ALT increase from the baseline (22 U/L) was not. At 3 days postinfusion, the AST and ALT levels (101 and 89 U/L, respectively) were still higher than the baseline, indicating acute toxicity, although no longer statistically significant. The levels of AST and ALT then decreased to the high end of their respective normal ranges: 91 and 80 U/L, respectively, at 4 days postinfusion. Comparing the peak of AST and ALT between the RMD treated and control groups demonstrated a significantly higher level AST (p < 0.05), whereas ALT was substantially, although insignificantly (p = 0.07) greater (**Figures 4A**).

Kidney biomarkers, urea nitrogen and creatinine, were scantily affected by RMD administration (**Figure 4B**). Prior to treatment, urea nitrogen and creatinine were 22 and 0.60 mg/dl, respectively. After RMD administration, the markers peaked at 35 and 0.77 mg/dl at 18 h postinfusion, respectively. Urea nitrogen increased above the normal range determined during our previous studies with RMs (8–25 mg/dl), while the level of creatinine remained within the normal range (0.5–1.2 mg/dl) and neither reached statistical significance. At 3 days postinfusion, creatinine returned to the baseline of 0.63 mg/dl, while urea nitrogen was still slightly elevated at 29 mg/dl and returned closer to the baseline level at 4 days postinfusion (25 mg/dl) (**Figure 4B**). The controls were also minimally impacted by anesthesia and saline administration (**Figure 4B**).

We assessed general toxicity by testing the levels of lactate dehydrogenase (LDH) and creatinine kinase (CK). Both LDH and CK increased after RMD infusion and neither returned to the baseline at 4 days postinfusion (**Figure 4C**). LDH increased from 702 to 1,562 U/L at 3 days postinfusion, while CK peaked at 7,445 U/L at 18 h postinfusion, from 367 U/L. These increases were substantially higher than the normal ranges of LDH [514 ± 187 U/L (61)] and CK (132–505 U/L, based on previous experiments). At 4 days postinfusion, CK (1,740 U/L) and LDH (1,323 U/L) declined, indicating a likely return to normal levels of both enzymes shortly thereafter. Although the only statistically significant change was CK at 18 h postinfusion (p < 0.05), these increases cannot be ignored. Similar to the liver markers, a significantly greater peak was observed in the LDH of the RMD treated RMs compared to the control group (p < 0.05), and yet a substantially, but insignificantly, higher peak was observed in the CK of the RMD treated RMs than control groups (**Figures 4C**).

To further investigate the differences in biomarker changes between the RMD treated and saline controls, we assessed the concentration of cortisol in plasma as a surrogate of stress. The cortisol levels increased in both groups after the infusion. However, whereas cortisol levels peaked in the saline control group at 6 h postinfusion and decreased toward the baseline levels from day 1 on, they remained elevated through 2 dpi in the RMs infused with RMD (**Figure S1**).

### Immune Cell Dynamics in Blood

Following RMD administration, total circulating lymphocytes experienced a transient, but significant decrease from 5,050 cells/μl at the baseline to 1,463 and 1,753 cells/μl at 12 h and 2 days postinfusion, respectively, p < 0.05 (**Figure 5A**). CD4^+^ T cells decreased from 2,518 cells/μl prior to treatment, to 402 cells/μl at 12 h postinfusion (p < 0.05), 496 cells/µl at 1 day postinfusion (p < 0.05) and were still decreased at 366 cells/μl (p < 0.05) through 2 days postinfusion, with a fast, strong recovery to 1,305 cells/μl at 5 days postinfusion (**Figure 5B**). Similarly, CD8^+^ T cells showed a substantial, significant decrease from 1,047 cells/μl at the baseline, to 204 cells/μl at 12 h postinfusion (p < 0.05), 178 cells/μl at 1 day postinfusion (p < 0.05) and 204 cells/μl at 2 days postinfusion (p < 0.05). CD8^+^ T cells started to recover back to 634 cells/μl at 5 days postinfusion, with near full recovery by 7 days postinfusion (741 cells/μl) (**Figure 5C**). B cells (CD3^−^CD20^+^) followed the same pattern as CD4^+^ and CD8^+^ T cells (**Figure 5D**). Conversely, neutrophils transiently increased from 3,273 to 14,287 cells/μl at 12 h postinfusion, well above the normal range of 6.2 ± 2.4 × 10^3^ cells/μl (61), followed by a rapid decline to 9,123 cell/µl at 1 day postinfusion and 2,427 cells/μl at 5 days postinfusion (**Figure 5E**). At no timepoint did the acute increase in neutrophils reach statistical significance.

Interestingly, the saline-infusion control group decreased in total lymphocytes after infusion, but the impact was subdued relative to RMD treated RM. In fact, both CD4^+^ (**Figure 5B**) and CD8^+^ T cells (**Figure 5C**) decreased to lower cell counts, while having higher baseline values, and took longer to recover in the RMD treated RMs. B cells declined in RMD treated RMs while there was no change in the control group (**Figure 5D**). Furthermore, the neutrophil dynamics of the saline-infusion control group had a much smaller increase compared to that of the RMD treated RMs and returned to baseline three days prior to the RMD treated group (**Figure 5E**).

To define the mechanism(s) of the CD4^+^ and CD8^+^ T cells decline after the RMD infusion, we first monitored the levels of apoptotic T cells by flow cytometry. The frequency of apoptotic CD4^+^ T cells decreased from 3.7% at the baseline to 1.8% after RMD administration (**Figure 6A**). In contrast, the frequency of apoptotic CD8^+^ T cells slightly increased from 2.1% at the baseline to 4.6% at 2 days postinfusion) (**Figure 6B**). The initial decrease in apoptosis was also observed in the saline control group, but the increased frequency of the apoptotic CD8^+^ T cells did not occur in controls (**Figures 6A, B**). Furthermore, the expression of T cell activation markers of CD69 and Ki-67 increased on both CD4^+^ and CD8^+^ T cells isolated from circulation from the RMD treated RMs by 1 day postinfusion (**Figure 7**), although none of the increases reached significance. This increase was in stark contrast to the saline controls which experienced no significant increase in the T cell immune activation levels (**Figure 7**).

### Downregulation of Surface Receptors of CD3, CD4, and CD8 After RMD infusion

Due to the massive, transient decrease in total counts of cells expressing CD3, including both CD4^+^ and CD8^+^ T cells, and the lack of corresponding increase in apoptosis, we conducted *ex vivo* RMD stimulation experiments on PBMCs and monitored changes in the expression of surface and homing markers on lymphocytes. Fresh PBMCs were incubated with various RMD doses (2, 10, and 20 ng/ml) for 1, 2, or 5 days. We observed a decrease trend of the expression of CD3, CD4, and CD8 surface markers, as measured by both individual cell expression (as mean fluorescence intensity) and total cells positive for the markers (% of cells). This decreased expression corresponded with the RMD dose and the duration in days of cell incubation with RMD (**Figure 8**). In the absence of RMD, CD3 expression increased from the baseline of 50% of cells to 56% at day 1, 66% at day 2, and 61% at day 5. In the presence of RMD, minimal changes occurred at day 1 for all dosage treatments. Then, CD3 expression decreased to 48%, 30%, and 32% at day 2 and decreased further to 20%, 16%, and 19% at day 5 for 2, 10, and 20 ng/ml, respectively (**Figure 8A**). These changes are staggering but did not reach statistical significance. Similar decrease trends of CD4^+^ (**Figure 8B**) and CD8^+^ (**Figure 8C**) expression were observed in time and dose dependent manners. Further, we noted complementary increased proportions of CD4^−^ CD8^−^ CD3^+^ T cells with increasing dosages of RMD (**Figure 8D**). However, the dependence on RMD concentration did not reach statistical significance for CD3^+^, CD4^+^, and CD8^+^ (p > 0.05), but the increase in CD4^−^ CD8^−^ CD3^+^ T cells did (p < 0.05). Our results thus suggest that the decreased expression of surface markers was due to extended RMD treatment rather than solely the amount of time in media.

### Homing Receptor Induction Suggests Migration of Lymphocytes to the Gut and LNs

To assess if the loss of the lymphocytes may be due to their migration to tissues, we measured the expression of various homing markers (α4β7, CCR4, CCR5, CCR7, and CCR9) on CD3^+^, CD4^+^, and CD8^+^ T cells in the same RMD stimulation experiments. α4β7 expression on CD4^+^ T cells at baseline was 8% and remained virtually unchanged without treatment, averaging 8% through day 2 (**Figure 9A**), and 7.5% at day 5. Under RMD treatment, α4β7 expression on CD4^+^ T cells increased to 9% (10 ng/ml) and 10% (20 ng/ml) at day 1 and to 16% (10 ng/ml) and 15% (20 ng/ml) at day 2, but surprisingly decreased to 2% (10 ng/ml) and 3% (20 ng/ml) at day 5, with no changes at any timepoint reaching significance. Contrary to CD4^+^ T cells, α4β7 expression on CD8^+^ T cells continuously increased with treatment (**Figure 9B**) from 7% of the baseline to 59% (2 ng/ml) (p > 0.05), 95% (10 ng/ml) (p < 0.05), and 91% (20 ng/ml) (p < 0.05) at day 5. In the absence of RMD, α4β7 expression on CD8^+^ T cells remained virtually unchanged regardless of incubation time. This demonstrates that the effect of RMD on α4β7 expression of on CD8^+^ T cells was due to both the incubation time and RMD concentration, p < 0.05.

CCR4 expression on CD4^+^ T cells was mostly unchanged (**Figure 9C**). CCR4 expression on CD8^+^ T cells dramatically increased from 6% of the baseline to 73% (2 ng/ml) (p > 0.05), 93% (10 ng/ml) (p < 0.05), and 90% (20 ng/ml) (p < 0.05) at day 5, which were much higher than 29% of untreated CD8^+^ T cells (p > 0.05) (**Figure 9D**). We found no statistical significance with CCR4 expression on CD4^+^ T cells, but did observe significance in the effects of incubation time (p < 0.05), yet not in RMD dosage on CCR4 expression on CD8^+^ T cells.

The changes in the expression of the gut homing marker CCR5 were the most discordant between CD4^+^ and CD8^+^ T cells. Regardless of RMD treatment, CCR5 expression on CD4^+^ T cells followed the similar decrease trend from 3% of the baseline to 2% (0 ng/ml) (p > 0.05), 1% (2 ng/ml) (p > 0.05), <1% (10 ng/ml) (p > 0.05), and <1% (20 ng/ml) (p > 0.05) at day 5 (**Figure 9E**). Conversely, on the untreated CD8^+^ T cells, CCR5 expression increased from 4% of baseline to 7% in the absence of RMD (p > 0.05), 59% with 2 ng/ml (p > 0.05), 93% with 10 ng/ml (p < 0.05), and 90% with 20 ng/ml (p < 0.05) at day 5, respectively (**Figure 9F**). The effect of incubation time was significant for CCR5 expression on both CD4^+^ (p < 0.05) and CD8^+^ T cells (p < 0.05), whereas only CD8^+^ T cell changes were significant for the effect of RMD concentration (p < 0.05).

In the absence of RMD, CCR7 expression decreased from 4% at the baseline to 2% at day 5 on CD4^+^ T cells and from 3% at the baseline to 2% at day 5 on CD8^+^ T cells. In comparison, CCR7 [LN homing (62)] and CCR9 [gut homing (63)] expression increased in both CD4^+^ and CD8^+^ T cells with RMD (**Figure 9**). CCR7 expression on CD4^+^ T cells peaked at day 5 after incubation with 20 ng/ml RMD (9%), p > 0.05 (**Figure 9G**). CCR7 expression on CD8^+^ T cells increased to a lesser extent (**Figure 9H**) but peaked at day 1 with 6% and 6.2% at 10 and 20 ng/ml, respectively. At day 5, CCR7 expression on CD8^+^ T cells (5%) with 20 ng/ml RMD was still more than that observed at the same timepoint with no treatment (2%). Although there is a trend toward increased CCR7 expression on CD4^+^ and CD8^+^ T cells with RMD treatment, neither the individual timepoint nor the effects of incubation time, nor RMD concentration, reached statistical significance.

The gut homing marker CCR9 (63) expression increased on both CD4^+^ and CD8^+^ T cells. CCR9 expression peaked at day 5 at 14% (10 ng/ml) and 13% (20 ng/ml) on CD4^+^ T cells. In the absence of RMD, CCR9 expression increased from 2% at the baseline, to 3% at day 5 (**Figure 9I**). These changes did not reach significance (p > 0.05). CCR9 expression on CD8^+^ T cells mirrored that on CD4^+^ T cells, with 4% of the baseline increasing to 8% (10 ng/ml) (p > 0.05) and 10% (20 ng/ml) (p > 0.05) at day 5. Meanwhile, CCR9 expression on untreated cells was of 3%, at day 5 (p > 0.05) (**Figure 9J**), further substantiating the increases observed after incubation with RMD. Statistical analysis of CCR9 expression with a mixed effects model demonstrated statistical significance with regards to incubation time in CD8^+^ T cells, p < 0.05, while RMD concentration was not statically significant (p = 0.0641). Such a statistical finding was not demonstrated with CD4^+^ T cells.

### CD3 Signal Increases in the Gut and LNs After RMD Administration

We further investigated the frequency of CD3^+^ T cells in the intestine and mesenteric and superficial LNs in the RMD treated RMs by IF on paraffin-embedded tissues. Using color thresholds to determine the area fraction positive for signal of stained CD3, we assessed changes in the relative amount of CD3^+^ T cells in tissues. In the superficial LNs, the amount of CD3^+^ T cells showed modest increases over 7 days, from an average of 30% of pretreatment to 40% and 41% area fraction, at 6 h and 7 days postinfusion, respectively (**Figures 10A–D**). Of note, we did not have tissue for IF from RM32 pretreatment, and the RM showed a decrease from 6 h to 7 days postinfusion, in contrast to the increases seen in the other two RMs. The mesenteric LNs were analyzed pretreatment and 1 day postinfusion, with only one animal (RM71) showing a moderate increase in CD3^+^ T cells by an area fraction change from 31% to 43%, respectively (**Figures 10E–G**). In the other two animals (RM32 and RM33) the CD3^+^ T cells area fraction remained virtually unchanged (p > 0.05). In the intestine, we observed an increase trend of CD3^+^ T cells in all three RMs from pretreatment to 1 day postinfusion (5.8% vs. 7.6%, respectively) (**Figures 10H–J**); yet, this increase did not reach statistical significance.

### RMD Administration Diminishes Cytotoxic T Lymphocyte Functionality

Previous studies reported conflicting data as to whether the functionality of CTLs is diminished by RMD administration (41, 64). Therefore, we performed functional assays on lymphocytes collected from superficial and mesenteric LNs and intestinal resections, focusing on the overall expression of the surrogate killing marker CD107a, along with granzymes A, B, and K, IFN-γ, and perforin with PMA and ionomycin stimulation (**Figure 11**).

All the markers of T cell functionality diminished upon RMD treatment, apparently confirming the results of previous studies (64). The CD107a expression decreased in the superficial LN from 1% at pretreatment to <1% at 2 days postinfusion. Similar changes were observed in the mesenteric LN (1% vs. <1% at 1 day postinfusion) and intestine (2% vs. 1% at 2 days postinfusion) (**Figure 11A**). Granzyme A decreased from 1% to <1% at 2 days postinfusion, 1% to <1% at 1 day postinfusion, and 2% to <1% at 2 days postinfusion in the superficial LNs, mesenteric LNs, and intestine, respectively (**Figure 11B**). Granzyme B decreased from 4% in the superficial LN to 1% at 2 days postinfusion, with similar changes in the mesenteric LN (5% vs. 2% at 2 days postinfusion) and intestines (11% vs. 3% at 2 days postinfusion) (**Figure 11C**). Granzyme K had similar decreases of 1% to <1% in the superficial LN at 2 days postinfusion, 2% to <1% in the mesenteric LN at 1 day postinfusion, and 1% to <1% in the intestine at 2 days postinfusion (**Figure 11D**). IFN-γ had moderate changes in its expression after RMD treatment. IFN-γ decreased in the superficial LN (4% vs. 1%) at 2 days postinfusion, mesenteric LN (5% vs. 3%) at 1 day postinfusion, and intestines (2% vs. 1%) at 2 days postinfusion (**Figure 11E**). Perforin also decreased in expression after RMD treatment. The superficial LN CD8^+^ T cells decreased perforin expression from 1% pretreatment to <1% at 2 days postinfusion, and the mesenteric LN and intestines mirrored these changes, decreased from 2% in the mesenteric LN to <1% at 1 day postinfusion and 1% in the intestines to <1% at 2 days postinfusion (**Figure 11F**). Thus, we found that RMD treatment resulted in a trend toward decreased cytotoxic functionality of CD8^+^ T cells in all three tissues assayed, indicated by decreases in the proportion of CD8^+^ T cells expressing these cytokines in response to PMA/ionomycin stimulation postinfusion (**Figure 11**). Further investigation into the multifunctionality of the CD8^+^ T cells demonstrated that there were substantial reductions in the percentage of CD8^+^ T cells expressing any combination of the cytokines in all three tissues as well as decreased proportions of cells producing higher combinations of cytokines (**Figure S2**).

## Discussion

RMD is a drug used in chemotherapy of cutaneous T cell lymphomas (65), which has garnered interest as a LRA, being considered as potentially one of the most potent HIV reactivators of the HDACi (40). We have previously reported that RMD is capable of reactivating latent SIV in post-treatment controllers (41). Here, we assessed the pharmacokinetics and safety profile of RMD by treating three SIV-naïve RMs with a 7 mg/m^2^ IV infusion over 4 h.

We report extensive tissue distribution of RMD from the blood in RMs with tissue: blood penetration ratios of ≥27 and ≥426 in the LNs and GI tract, respectively. Polyexponential clearance from the plasma that is typically indicative of extensive tissue distribution has been previously described for RMD in humans (48, 66). Consistent with these observations, we also observed biphasic plasma elimination. Our estimated terminal elimination half-lives are longer than those reported in humans (~3.5 h) (66), and rhesus macaques (67). However, both of these studies appear to calculate their terminal elimination half-lives based on the early distribution phase, which limits the accuracy of the terminal clearance of RMD in those studies. Similarly, in our study, RMD exhibited an early distribution half-life of approximately 3–6 h. In fact, clinical studies providing estimates of RMD’s terminal elimination half-life (6.6–19 h) (48) are consistent with our findings of ~15.3 h. This is likely due to the extended timepoints that were measured and give a better depiction of the terminal elimination half-lives.

We also report that, in contrast to the relatively rapid RMD clearance from circulation, RMD persists in the LNs and intestine significantly longer, and is still detectable 10 days postinfusion in these tissues. However, we currently do not understand the mechanism of persistence. Because we measured the prodrug form of RMD, it is possible that the maintained concentrations are due to either greater distribution to the tissue compartments, reduced conversion of the RMD to the active, reduced form, trapping of RMD between the extracellular and interstitial spaces, or a combination of these. As it is, the prolonged RMD half-life and the ability to quantify RMD at 10 days postinfusion in tissues compared to plasma is of interest, as the HIV reservoir is known to persist in the LNs and GALT (15, 68, 69) and viral diversity during recrudescence of infection post-ART interruption suggests multiple sources of reactivation (70). Thus, the sustained concentration of prodrug RMD in the intestinal and lymphoid tissues addresses this issue and suggests that RMD is a viable drug for reactivation studies. In fact, the plasma concentrations of RMD given at 7 mg/m^2^ were greater than that found to readily reactivate HIV *in vitro* (5 nM) (43) through 8 h postinfusion [5.16 nM (2.79 ng/ml)].

The brain is also a key HIV reservoir (71–73). However, we did not collect central nervous system (CNS) tissue, nor cerebral spinal fluid (CSF), an established surrogate for testing drug penetration of the blood-brain barrier (74), to test potential penetration of RMD into the brain. Our rationale was that previous studies have demonstrated that RMD has poor penetration into the CSF, with only 2% the AUC of CSF/plasma, therefore suggesting poor penetration into the brain (67). At 2 h postinfusion, the plasma concentration was 19.9 ng/ml (37.6nM). Assuming a 2% penetration, this would have equate to 0.73nM in the CSF, which is not a strong enough concentration to reactivate HIV *in vitro* (43). Further compounding the lack of penetration is that HIV strains isolated from the CNS were shown to contain polymorphisms in the LTR which resulted in reduced abilities to reactivate, including when treated with RMD (75). Thus, we reasoned that it is unlikely that RMD would reactivate HIV/SIV in the CNS at the given dose of 7 mg/m^2^ and we did not focus on the brain in this study.

Interestingly, we observed an increased concentration of RMD in the plasma of RM32 *vs*. the other two RMs, which was accompanied by a lower intestinal concentration at 4 and 48 h. These are strikingly different concentrations: 47 ng/ml vs. 8 and 5 ng/ml at 2 h postinfusion in the plasma and 354 ng/g vs. 3,932 and 3,666 ng/g at 4 h postinfusion in the intestines of RM32, RM33, and RM71, respectively. Upon further investigation, we noted that RM32 had the greatest lymphopenia and subsequently the least immune activation with a slight delay in immune rebound. However, blood chemistries did not show a significant difference in enzyme changes. We also noted a more reduced histone acetylation in RM33 relative to the other two RMs, whereby 1 dpi was the most notable increase. Plasma and tissue RMD concentrations were not lower relative to the other RMs, however, suggesting a different unknown mechanism for the reduced acetylation.

We next assessed the RMD toxicity in the SIV-naïve RMs. By performing blood chemistries, we assessed the levels of markers for hepatotoxicity (AST and ALT), nephrotoxicity (urea nitrogen and creatinine), and general toxicity (LDH and CK). The levels of urea nitrogen and creatinine were the least changed within the three categories, with a minimal increase that was still within the normal range for RMs (61), indicating that RMD has little to no nephrotoxicity in RMs. It is likely that the minimal elevations seen in urea nitrogen and creatinine are due increased protein turnover rate resulting from biopsy procedures. This is further supported by the results in the control group which did not have biopsy procedures conducted and had even fewer increases in urea nitrogen and creatinine than the RMD group. AST and ALT increased to levels that were well above the normal range and returned to normal by 4 days postinfusion, suggesting moderate liver toxicity which was not observed in the saline-infusion group. The increases seen in CK and LDH were transient, both biomarkers returning to baseline by 4 days postinfusion, indicating transient systemic toxicity in line with the observed loss of lymphocytes from the periphery. Another explanation for the increases in AST, ALT, CK, and LDH could also be musculoskeletal damage. With the long sedation events and biopsies at the beginning of experimentation, it is possible that we are observing damage from these procedures, ketamine-induced rhabdomyolysis (76), or stress. The increase in CK and the lack of LDH increase in the control group is in line with previous studies indicating that isoflurane can increase serum CK, while having no significant effect on LDH (77). Although the elevated cortisol corresponds to the increase in CK, we did not observe a corresponding increase in AST, ALT, and LDH that we would have expected if stress were a major contributing factor to the toxicity (78). Therefore, the substantially raised liver enzymes, CK, and LDH relative to the saline control group is best explained by a mixture of toxicity as a result of RMD treatment, biopsy-mediated rhabdomyolysis, and a small contribution from stress, with some of the CK changes due to isoflurane anesthesia. Overall, we report that RMD administration should be carefully monitored if given and using a dosage of 7 mg/m^2^ or greater risks liver damage in RMs.

Previous studies have reported an immediate and brutal lymphopenia after RMD treatment (42). In contrast to what is seen in a traditional bone marrow transfer, where regeneration of T cells occurs in approximately 60 days (79), the T-cell rebound after RMD administration was very rapid, starting by 2 days postinfusion, and resulting in a near-full restoration as early as 5 days postinfusion for CD8^+^ T cells and 7 days postinfusion for CD4^+^ T cells. However, the saline control group had less drastic, transient lymphopenia that recovered days earlier, in line with previous studies demonstrating immune suppression after isoflurane anesthesia with increased cortisol (80, 81). These results thus suggest that the isoflurane anesthesia used played a small role in the initial loss of lymphocytes from the periphery. Moreover, the fractions of apoptotic CD4^+^ and CD8^+^ T cells were scantily affected by RMD treatment and the Ki-67 expression, a marker of lymphocyte proliferation, on CD4^+^ and CD8^+^ T cells was not massively increased during the time of lymphocyte recovery. Interestingly, immune cell fluctuations after recovery, e.g., day 15, were concomitant with a secondary increase in CD69 and Ki-67 expression and also seen in other studies (41, 43), although the exact mechanism of the delayed response is not known. Further, upon *in vitro* treatment with RMD, we observed the decrease in surface expression of CD3, CD4, and CD8 with complementary increases in double negative CD3^+^ T cells. This suggested that the lymphocyte populations were not decreasing only by cell death, but also by a combination of downregulation of T cell surface markers, upregulation of homing markers, and subsequent migration from the periphery to the intestines and lymphatics along with retention in lymphatics. This does not necessarily imply the lack of cell death. In fact, the massive increase in segmented neutrophils immediately after treatment concomitant with the loss of lymphocytes is indicative of an immune response to damage-associated molecular patterns. However, changes in the expression of cell surface markers may occur as a result of activation, as previously demonstrated with T-cell stimulation by the phorbol ester PMA (82); meanwhile, increased CD69 expression follows lymphocyte retention in lymphoid organs (83). Both of these mechanisms may explain the changes observed after RMD infusion. In line with this theory, we observed an immediate, transient increase in CD69 after infusion. When RMD was administered *in vitro*, CD3, CD4, and CD8 expression decreased while homing markers responded differently from each other between CD4^+^ and CD8^+^ T cells. Increases of CCR7 (LN homing marker) and CCR9 (gut homing marker) expression were observed for both CD4^+^ and CD8^+^ T cells, although only the RMD effect on CCR7 expression on CD8^+^ T cells reached significance. Additionally, we found that the CD8^+^ T cells expressed massive, significant increases of CCR4, CCR5, and α4β7 expression with RMD treatment. Thus, the changes in expression of CD69 and homing markers, together with the increase in CD3^+^ T cells in the gut and LNs, further suggest an additional mechanism in which peripheral lymphocyte reduction was achieved through a combination of retention and migration to the gut and LNs. Together, these data suggest that the cause of the transient loss of CD4^+^ and CD8^+^ T cells in the periphery is due to a combination of isoflurane anesthesia causing stress that leads to the initial loss, with the major contribution from subsequent effects of RMD treatment: (i) downregulation of lymphocyte surface markers, (ii) retention of lymphocytes in the lymphatics, (iii) upregulation of specific homing markers, and (iv)subsequent migration of peripheral lymphocytes to the intestines and LNs, and minimal apoptosis, in agreement with the postulated reasoning in our previous report (41).

RMD has been reported to have a negative effect on the cytotoxic lymphocyte response (64), but this finding has been contradicted (41). We found that with RMD treatment, CD8^+^ T cells expressed fewer markers of cytotoxic functionality and lost some of the multifunctionality (CD107a, Granzymes A, B, and K, IFN-γ, and perforin). Importantly, the expression of these markers was also hindered by the RMD treatment when the cells were stimulated with PMA and ionomycin. Although these data seem to be in agreement with RMD having a negative effect on the CTL responses, they did not reach significance. However, our results must be taken with caution, as the PMA and ionomycin treatment is not an exact replica of antigen response. Nonetheless, our data suggest that RMD may work the best as an HIV therapeutic in combination with an immune activator, such as a PD-1 or CTLA-4 blockade, or a Treg depletion agent (20), to supplement the cell-mediated immune response for eliminating infected, reactivated cells.

Although the “shock and kill” approaches for the HIV cure are moving away from HDACi and toward novel agents, such as STING agonists (84) and SMAC mimetics (84–86), our results suggest that RMD may still hold a place within HIV cure. We demonstrated that the observed lymphopenia postinfusion is due to a combined RMD effect, consisting of (i) apoptosis; (ii) downregulation of cell surface markers CD3, CD4, and CD8; (iii) upregulation of homing markers α4β7, CCR7, and CCR9; and (iv) migration from the periphery to the gut and LNs. As such, our results show that, despite RMD’s acute toxicity and hindrance of T lymphocyte functionality, RMD’s potent HDAC inhibition, coupled with its prolonged pharmacokinetics in tissue, makes it a drug that would benefit from being used in combination with another therapeutic agent, particularly one with minimal liver toxicity that enhances the cell-mediated immune response, as an effective LRA, in HIV cure.

## Data Availability Statement

The raw data supporting the conclusions of this article will be made available by the authors, without undue reservation.

## Ethics Statement

The animal study was reviewed and approved by the Institutional Animal Care and Use Committee (IACUC) of the University of Pittsburgh (protocol 15045866).

## Author Contributions

AJK, CA, IP, and AK designed and oversaw the study. AJK, CX, and RS contributed to sample processing. TD provided veterinarian care, administrated the drugs, and collected samples. AJK and EB-C performed flow cytometry experiments and analyses. AJK and RS performed histologies, IHC stainings, and quantifications. MC and AK performed pharmacologic analyses. EB-C and AJK performed functional studies. AJK performed statistical analyses, generated figures, and participated in the data analyses. IP performed histological diagnoses. AK and MC helped with study design, data interpretation, and discussion. IP, CA, and AJK wrote the manuscript. CX, MC, EB-C, and RS edited the manuscript. All authors contributed to the article and approved the submitted version.

## Funding

This work was funded by grant R01 AI119346 (CA) from the National Institutes of Health (NIH)/National Institute of Allergy and Infectious Diseases (NIAID) and also from grants R01DK113919 (IP/CA), R01DK119936 (CA), RO1 HL117715 (IP), R01 HL123096 (IP) from the National Institute of Diabetes and Digestive and Kidney Diseases (NIDDK) and National Heart, Lung and Blood Institute (NHLBI). AJK was supported in part by the NIAID and T32 grants Immunology of Infectious Diseases (IID) (AI060525) and Pitt AIDS Research Training (PART) grant (AI065380). Funders had no role in study design, data collection and analysis, decision to publish, or preparation of the manuscript. The content of this publication does not necessarily reflect the views or policies of the Department of Health and Human Services, nor does mention of trade names, commercial products, or organizations imply endorsement by the U.S. Government.

## Conflict of Interest

The authors declare that the research was conducted in the absence of any commercial or financial relationships that could be construed as a potential conflict of interest.
