# Supplementary material for: Pharmacokinetics and Immunological Effects of Romidepsin in Rhesus Macaques
Source: Front Immunol. 2020 Dec 11;11:579158. doi: 10.3389/fimmu.2020.579158 (PMC7759686; doi:10.3389/fimmu.2020.579158)
Supplement: Supplementary file 1 [file DataSheet_1.docx]

**Supplemental Materials**

**Supplemental methods.**

**Cortisol ELISA.** To assess the levels of stress associated with the different animal procedures used, frozen plasma was thawed, and dilutions of either 1:16 to 1:50 were used to measure the circulating cortisol levels with a cortisol ELISA kit (ENZO, Farmingdale, NY, USA), as per manufacturer instructions. The results were read at 405 nm on a Ultrospec 2100 Pro (Amersham Biosciences, Little Chalfont, UK) and results interpolated by Prism 8.4.3 (GraphPad Software, Inc., San Diego, CA) using 4 parameter logistic curve fitting.

**Supplemental figures.**

**
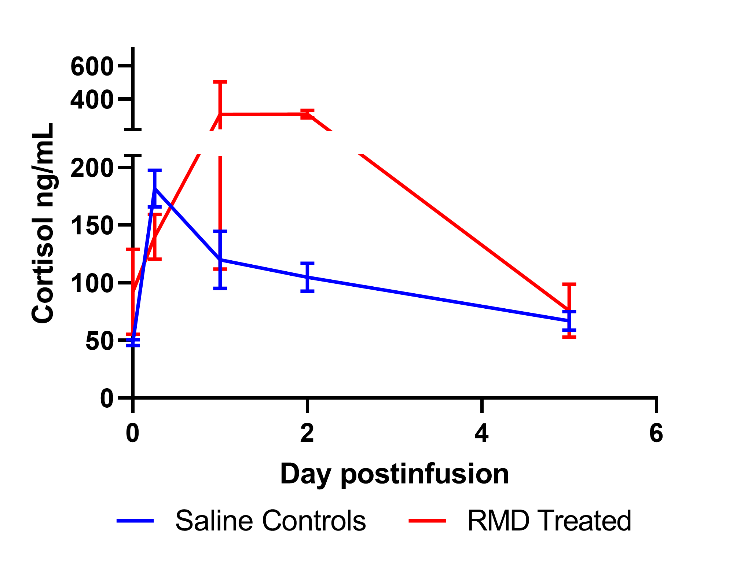
**

**Figure S1. Cortisol concentrations in plasma after RMD and saline infusions.** Cortisol ELISA was performed with plasma at timepoints pre, 6 hpi, 1, 2, and 5 dpi from both RMD treated and saline control groups. Data is shown as mean ± SEM, n=3 per group.


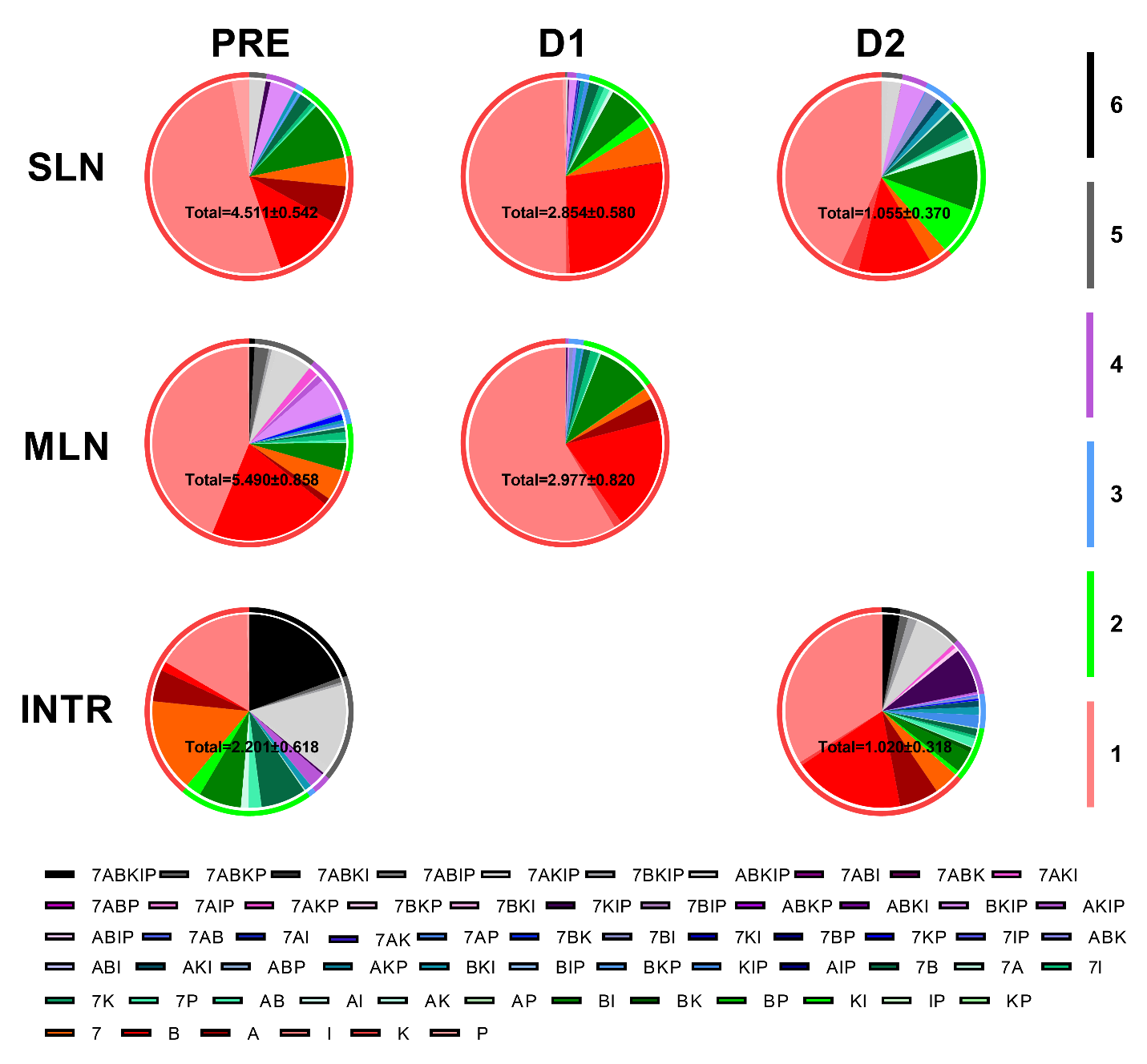


**Figure S2. Multifunctional CD8^+^ T cells after RMD administration.** Functional assays were performed for cytotoxic T lymphocyte markers of functionality, CD107a (7), Granzymes A (A), B (B), and K (K), IFN-γ (I), and perforin (P) in PMA and ionomycin stimulated CD8+ T cells from: SLN at pre 6 hours postinfusion and 2 days postinfusion, MLN at pre and 1 day postinfusion, and INTR at pre and 2 days postinfusion. Inner circle is colored by individual combinations as shown in the lower legend and the number denotes total percentage of CD8^+^ T cells expressing any combination of the stated cytokines ± SEM, n=3 per group. The ring shows the proportion of cells presenting combinations of 1, 2, 3, 4, 5, or all 6 cytokines tested as designated by the side legend. SLN: Superficial LN; MLN: Mesenteric LN; INTR: Intestinal resections.
